# Integrated surgical treatment: a new model for treating secondary extremity lymphedema based on algorithms

**DOI:** 10.3389/fonc.2025.1676803

**Published:** 2026-01-12

**Authors:** Junzhe Chen, Zongcan Chen, Xiangkui Wu, Hai Li, Shune Xiao, Zairong Wei, Yixin Zhang, Chengliang Deng

**Affiliations:** 1 Department of Burns and Plastic Surgery, Affiliated Hospital of Zunyi Medical University, Zunyi, Guizhou, China; 2 The Collaborative Innovation Center of Tissue Damage Repair and Regeneration Medicine, Zunyi Medical University, Zunyi, Guizhou, China; 3 The Department of Plastic and Reconstructive Surgery, Shanghai Ninth People’s Hospital, Shanghai JiaoTong University School of Medicine, Shanghai, China

**Keywords:** lymphedema, surgery, anastomosis, lymph node, liposuction

## Abstract

Secondary extremity lymphedema (SEL) is a chronic and progressive disorder resulting from impaired lymphatic drainage, most commonly following oncologic interventions such as breast or gynecological cancer surgery. Characterized by the accumulation of lymphatic fluid, progressive inflammation, adipose hypertrophy, and tissue fibrosis, SEL poses significant therapeutic challenges, particularly in its advanced stages. While conservative management remains the first-line treatment for mild cases, surgical intervention becomes essential in moderate to severe disease. Surgical approaches are generally categorized into physiological procedures, which aim to restore lymphatic continuity (e.g., lymphaticovenous anastomosis [LVA], vascularized lymph node transfer [VLNT]), and excisional techniques, which remove fibrotic and adipose tissue (e.g., liposuction, Charles procedure). Increasingly, integrated strategies combining physiological and excisional methods have demonstrated superior outcomes by targeting both fluid and solid components of the disease. However, multiple challenges remain, including the identification of functional lymphatic vessels, donor site morbidity, variability in long-term outcomes, and a lack of standardized surgical algorithms. Emerging evidence supports a component-based, individualized surgical framework tailored to disease severity, pathological tissue composition, and lymphatic functionality. Combined approaches such as the “3L” strategy (LVA, VLNT, and liposuction) have shown promise in enhancing volume reduction, minimizing infection risk, and improving quality of life. This review synthesizes recent advancements in SEL surgery and proposes a practical decision-making algorithm, the ISTL algorithm, which integrates clinical evaluations, imaging diagnostics, and surgical interventions for personalized treatment planning and improved long-term surgical outcomes.

**Clinical Trial Registration:**

This study was registered at the China Clinical Trial Registration (www.chictr.org.cn) with the registration number NCT06920732.

## Introduction

Secondary extremity lymphedema (SEL) is a chronic, progressive condition arising from impaired lymphatic drainage, commonly due to infection, trauma, or malignancy. This impairment leads to the accumulation of lymphatic fluid in the interstitial spaces and subsequent pathophysiological changes (1). In early stages, SEL presents with inflammation and edema, progressing to hypertrophic adipose deposition and tissue fibrosis in more advanced stages (2). With the increasing incidence of cancer, tumor-related lymphedema has become the predominant form of SEL, most frequently involving the upper limbs following breast cancer surgery and the lower limbs after gynecological procedures, with reported incidence rates of 8.4–21.4% and 20–60%, respectively (3, 4).

For mild SEL, conservative approaches—particularly comprehensive decongestive therapy (CDT)—are the mainstay. However, recent studies and clinical observations indicate that CDT can also be effective in moderate and severe cases, especially when initiated early and with proper patient compliance. While surgical intervention is often necessary in advanced stages, CDT continues to offer significant benefits for many patients, even those with moderate and severe lymphedema, as a primary or adjunctive therapy before opting for surgery (5).

**Figure 1:**
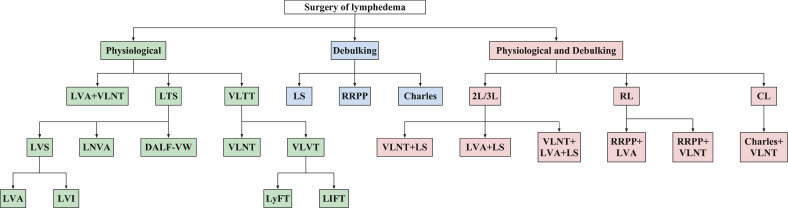
Surgery classification types of SEL. LVA, lymphovenous anastomoses; VLNT, vascularized lymph node transfer; LTS, lymphatic tissue shunts; LVS, lymphaticovenous shunts; LVI, lymphaticovenous implantation; LNVA, lymph node to vein anastomosis; DALF-VW, dermal-adipose lymphatic flap venous wrapping; VLTT, vascularized lymphatic tissue transfer; VLVT, vascularized lymph vessel transfer; LyFT, lymphatic flow-through flap; LIFT, lymph interpositional flap transfer; LS, liposuction; RRPP, radical reduction with preservation of perforators.

Surgical techniques are broadly divided into physiological methods, which restore lymphatic drainage, and debulking methods, which address excess fibrotic and adipose tissue (**Figure 1**). Each surgical method has distinct indications and limitations, underscoring the need for integrated surgical strategies. In recent years, combined surgical approaches have increased, primarily categorized into physiological integrated physiological surgery and physiological integrated debulking surgery. Lymphaticovenous anastomosis (LVA) offers near-immediate relief by diverting excess fluid into the venous system but requires functional lymphatic vessels, which many patients lack. In contrast, vascularized lymph node transfer (VLNT) does not depend on functional lymphatic vessels, though its therapeutic effect is delayed. While surgical techniques such as LVA and VLNT are crucial for advanced stages, CDT continues to play an important role in managing moderate and severe cases, especially as a complementary treatment before surgery or as part of a combined approach to optimize outcomes.

Combining LVA and VLNT achieves both short- and long-term benefits and retains the option of secondary surgical intervention if LVA fails (6–8). Despite these advantages, physiological integrated physiological methods may overlook the solid component (hypertrophic fat and fibrotic tissue) of lymphedema, particularly in severe SEL. While physiological techniques primarily address excess fluid, excisional procedures remain essential for removing solid tissue. Fillon et al. (9) have proposed that combining physiological and excisional surgeries may yield superior outcomes by simultaneously targeting both fluid and solid components, thus reducing swelling, minimizing reliance on compression therapy, and improving quality of life(QOL). Nonetheless, significant challenges persist, including optimal sequencing of procedures, the need for standardized algorithms, and decisions regarding simultaneous or staged operations. The objective of this study is to review the current surgical management of SEL and propose a novel algorithm that accounts for disease severity, involved anatomical regions, lymphatic vessel functionality, and clinical presentation. This framework aims to guide surgical planning and decision-making in lymphatic surgery systematically.

## Treatment of SEL with lymphatic tissue shunts

Lymphatic tissue shunts (LTS) refer to surgical connections that link lymphatic tissue to the venous system, with the goal of enhancing lymphatic fluid drainage. These procedures include LVA, lymphaticovenous implantation (LVI), dermal-adipose lymphatic flap venous wrapping (DALF-VW), and lymph node to vein anastomosis (LNVA). Among the most performed surgeries for lymphedema is LVA, a microsurgical technique that directly connects lymphatic vessels to veins, allowing lymphatic fluid to flow into the venous circulation. In 1969, Yamada had begun its clinical application for lymphedema. In 2000, Koshima et al. (10) advanced LVA by employing supermicrosurgical techniques to anastomose lymphatic vessels as small as 0.3–0.8 mm with adjacent superficial subcutaneous veins. The advantages of LVA are significant; it is minimally invasive, can be performed under local anesthesia, and exhibits rapid effectiveness (with observable limb decongestion during the procedure), resulting in satisfaction in the early stages. Numerous meta-analyses have demonstrated that LVA effectively reduces swelling, alleviates pain, and lowers infection rates in patients with lymphedema, and it may also aid in preventing the onset of the condition (11, 12).

Despite more than fifty years of advancement, disputes persist, awaiting resolution. Firstly, the ideal quantity of lymphatic vessels required for anastomosis remains unclear. A debate continues regarding the “quantity” versus “quality” of these anastomoses. Initial studies by Koshima et al. (10) indicated that connecting approximately 10 lymphatic vessels could significantly alleviate symptoms of lymphedema, while other research has demonstrated satisfactory outcomes with as few as 1–2 functioning lymphatic vessels (13). More recent investigations have introduced the concept of “lymphosome”—specific lymphatic regions that do not communicate with one another—suggesting that each lymphosome may require separate anastomoses to achieve optimal results (14, 15). Historically, it was believed that individuals with severe lymphedema derive limited benefits from LVA due to extensive tissue fibrosis and damage to lymphatic vessels. However, Han et al. (16) challenged this perspective by demonstrating that even individuals in advanced stages of lymphedema can benefit from LVA, provided that functional lymphatic vessels are present. Collectively, these findings indicate that patients may achieve positive outcomes from LVA when enough anastomoses are performed across various lymphosomes with functional lymphatic vessels.

Secondly, identifying functional lymphatic vessels remains a significant challenge. Although Johnson et al. (17)suggested that lymphatic vessels exceeding 0.5 mm in diameter may be optimal for functionality in lower limb lymphedema LVA, reliably identifying these vessels continues to pose difficulties. Common intraoperative techniques, such as infrared imaging and fluorescent microscopy, are frequently utilized; however, each method has inherent limitations. Infrared imaging with indocyanine green(ICG) suffers from restricted depth penetration, complicating the visualization of linear lymphatic vessels in cases of moderate to severe lymphedema. Conversely, fluorescent microscopy, like the Zeiss K900, requires the exposure of lymphatic vessels for effective observation (18). High-frequency ultrasound, as a noninvasive and innovative method, is effective in identifying lymphatic vessels without relying on contrast injection. However, it presents a steep learning curve that can hinder many surgeons from achieving proficiency, thereby limiting its broader implementation (19). In contrast, contrast-enhanced ultrasound (CEUS) has emerged as a promising technique, offering a more straightforward and accessible method for differentiating functional lymphatic vessels, which enhances its applicability in clinical practice (20). Nevertheless, a significant drawback of CEUS is the transient visibility of lymphatic vessels; if vessels are not discernible during the initial imaging, multiple contrast injections may be necessary to achieve adequate visualization. Despite these advancements, the consistent identification of functional lymphatic vessels remains complex and continues to be an active area of research.

Lastly, the long-term effectiveness of LVA remains a subject of ongoing debate. In standard physiological conditions, lymphatic capillary pressure typically ranges from approximately 0 to 8 mmHg (21), while superficial venous pressure is generally between 20 and 25 mmHg (22). In instances of lymphedema, the outflow of lymphatic fluid is obstructed, leading to an abnormal increase in lymphatic pressure. LVA promotes the drainage of fluid based on this pressure differential, facilitating the decongestion of the affected limb. However, once adequate decongestion is achieved, lymphatic pressure may equal or fall below venous pressure, potentially resulting in retrograde flow at the anastomosis site and causing recurrent swelling (23). To address this issue, various modified LVA techniques have been introduced, including Venturi-type (24), Pi-shaped (25), lymphaticolymphatic anastomoses (26), and lambda-shaped LVA (27). Nevertheless, further research is required to validate their long-term effectiveness.

In 1981, Degni first introduced LVI, a technique involving the direct insertion of lymphatic vessels with surrounding adipose tissue into a vein (28). A modified approach—the “octopus” technique, enabling the simultaneous implantation of multiple lymphatic vessels into a single vein (29). Compared to LVA, which requires precise end-to-end suturing, LVI offers several advantages: it obviates the need for microsurgical anastomosis, reducing technical complexity and operative time; it eliminates the need for size matching between lymphatic vessels and veins, increasing surgical flexibility; and it allows for multiple vessel implantations, potentially enhancing lymphatic drainage. However, LVI carries notable risks. The use of larger veins, inclusion of perivascular tissue, and absence of precise anastomosis may elevate the likelihood of postoperative thrombosis and embolic events.

In 2023, DALF-VW was introduced by Yamamoto et al. (30), specifically targeting individuals suffering from advanced lymphedema primarily associated with fibrosis. This method employs capillary lymphatic vessels located within a dermal flap to enhance lymph drainage, incorporating venous valves to prevent retrograde flow. Furthermore, DALF-VW can be performed under local anesthesia and does not require high-level microsurgical expertise, resulting in a significant reduction in operative duration and minimizing patient trauma.

In 2013, Olszewski (31) introduced the concept of LNVA, a technique that involves the partial excision of a lymph node, which is subsequently connected to a vein to enhance lymphatic drainage into the venous system. However, this method often requires the severing or disruption of the majority of afferent and efferent lymphatic vessels, which can limit its overall drainage effectiveness. In 2021, Pak proposed a modification to LNVA that involves puncturing and dilating the lymph node prior to linking it to the vein at the site of puncture, thereby improving drainage efficiency (32). This revised LNVA method preserves a greater amount of lymph node tissue and its function, leading to improved drainage outcomes. Despite this advancement, late-stage lymphedema is often characterized by pathological alterations that result in lymph node degeneration and loss of function, complicating the preoperative identification of functional lymph nodes and restricting the broader application of LNVA.

LTS is a widely accepted physiological strategy for managing lymphedema, supported by recent advancements in microsurgical technologies. In the early stages of lymphedema, which are primarily characterized by fluid retention, LTS can be highly effective on its own. However, in moderate to late-stage lymphedema, where there is an accumulation of fat and fibrosis, the standalone effectiveness of LTS diminishes, although some degree of limb decongestion may still be achievable. Ongoing challenges, such as postoperative venous reflux, compromised quality of lymphatic vessels in advanced stages, and the complexity of surgical procedures, impede the full effectiveness of LTS.

## Vascularized lymphatic tissue transfer for lymphedema

Vascularized lymphatic tissue transfer (VLTT) refers to the procedure of transferring a vascularized flap that includes a functional lymphatic network to address lymphedema. This approach encompasses both VLNT and vascularized lymph vessel transfer (VLVT). In 1982 the first clinical application of VLNT for lower extremity lymphedema conducted by Cloudius et al (33, 34). This method involves the transplantation of lymph nodes along with adjacent lymphatic tissue and their blood supply into regions with obstructed lymphatic drainage. The transplanted lymph nodes are capable of secreting lymphangiogenic growth factors, which promote capillary lymphangiogenesis and aid in reconstructing lymphatic pathways (35). Additionally, they can absorb nearby lymph fluid and direct it into the venous system via lymphatic-venous connections found within the nodes, thereby alleviating swelling in the limbs. Given that VLNT does not rely on the presence of functional lymphatic vessels in the recipient region, it is applicable for all stages of lymphedema, particularly in advanced cases characterized by severe fibrosis where LTS is not feasible. Consequently, VLNT has gained recognition as the preferred option for treating late-stage lymphedema (36). Multiple systematic reviews have demonstrated that VLNT effectively diminishes limb swelling, reduces the incidence of skin infections, lessens reliance on compression therapy, and enhances the QOL for individuals suffering from lymphedema (37, 38).

Despite more than four decades of development, VLNT faces several challenges:(1)Donor Site morbidity and Limited Donor Availability: Common VLNT donor sites include superficial locations such as the groin, lateral thorax, submental, and supraclavicular regions, as well as visceral sites like the omentum and mesentery (39). Harvesting from superficial sites may damage lymphatic and neurovascular structures, potentially causing lymphatic leakage, sensory deficits, or even iatrogenic lymphedema, particularly when taken from the groin or lateral thorax. Although reverse lymphatic mapping can reduce the risk of donor site lymphedema, it does not completely prevent it (40). Harvesting visceral lymph nodes requires multidisciplinary collaboration, adding complexity to the procedure. (2) Optimal number of lymph nodes for transplantation: The precise number of lymph nodes required for effective treatment remains inadequately defined (41). Some studies suggest that transplanting 2–3 lymph nodes is more effective in reducing limb circumference compared to using a single node (42). However, no significant difference in the incidence of skin infections has been observed when comparing single to multiple lymph node transplants. While additional nodes may enhance efficacy, collecting an excessive number can result in increased morbidity at the donor site. (3) Impact of Age on Lymph Node Function: The functionality of lymph nodes tends to decline with age due to degeneration and atrophy. It is crucial to investigate the long-term outcomes of VLNT in older patients to determine the effectiveness of this procedure within this demographic (43). (4) Optimal Recipient Site for VLNT: A consensus on the most appropriate recipient site for VLNT has yet to be achieved (44). Research indicates no significant difference in drainage functionality and therapeutic effectiveness between placements in the proximal versus distal limb (38). Nonetheless, some studies have reported favorable outcomes with mid-limb placements (45).(5) Time to Therapeutic Effect: The therapeutic benefits of VLNT typically emerge after a certain delay. Due to the limited capacity for lymphatic drainage in the recipient area prior to transplantation, it generally takes about six months for lymphatic flow restoration to occur.

In 2015, Koshima and colleagues (46) introduced the VLVT technique, which employs the first dorsal metatarsal artery as its vascular source. This method involves the transfer of lymphatic vessels along with the adjacent lymphatic tissue, while excluding lymph nodes, thereby reducing the likelihood of lymphedema at the donor site. Following its introduction, a significant question emerged regarding whether the lymphatic vessels in the affected limb should be directly connected to the transplanted flap. This inquiry has led to the development of two distinct concepts: Lymph Interpositional Flap Transfer (LIFT) and Lymphatic Flow-Through Flap (LyFT).

Yamamoto et al. (47) introduced the LIFT methodology, which is based on the concept of lymph axiality. They proposed that when local and transferred lymphatic vessels are closely situated, they may reconnect spontaneously, thereby promoting lymphatic drainage without the need for direct anastomosis. This approach has the potential to reduce both the duration of surgery and its technical challenges. However, Yamamoto et al. (48) also noted that the presence of non-lymphatic tissue between the ends of lymphatic vessels could impede spontaneous reconnection, leading to the failure of the LIFT technique. In contrast, Summa et al. (49) presented the LyFT technique, which advocates for direct lymphatic-venous anastomosis between the lymphatic vessels of the affected limb and those in the transferred flap. Although the LyFT technique requires meticulous microsurgical anastomosis, it ensures lymphatic continuity, which is likely to yield improved outcomes in cases where spontaneous reconnection is obstructed by non-lymphatic tissues (50). Both LIFT and LyFT offer distinct advantages, and further comprehensive long-term studies are essential for evaluating their effectiveness in clinical applications. An additional area of debate within VLVT pertains to the optimal volume of lymphatic tissue for transplantation. While it is generally accepted that transferring a larger volume of lymphatic tissue may enhance outcomes, there is currently no standardized quantitative guideline.

The superficial circumflex iliac perforator (SCIP) flap is the most commonly used option for VLVT, favored for its rich lymphatic vessel content and consistent pathways in the iliac and inguinal regions (51). The anterolateral thigh flap also serves as a viable alternative, valued for its versatility and the surgical familiarity it offers, which facilitates broader clinical application without the need for specialized training (52). Although the precise mechanism of VLVT remains unclear, it is hypothesized to involve either intrinsic lymphatic vessel pumping or the spontaneous formation of lymphatic anastomoses between the flap and recipient site. Unlike VLNT, VLVT preserves donor-site lymph nodes, reducing the risk of iatrogenic lymphedema; however, it lacks the potential immunological benefits conferred by VLNT. Despite promising early outcomes, current evidence on VLVT is limited to small case series, underscoring the need for larger, controlled studies to assess its long-term efficacy. Both VLNT and VLVT offer promising alternatives across lymphedema stages—particularly for patients unsuitable for LVA—and expand the therapeutic arsenal for managing chronic lymphedema.

## Charles procedure and RPPP in lymphedema treatment

First described by Charles in 1901 (53), the Charles procedure is recognized as the pioneering surgery for lymphedema. This technique involves the open removal of diseased tissue and skin above the deep fascia, followed by the application of skin grafts or reimplantation onto the exposed areas to thoroughly eliminate pathological tissue (54). In cases of advanced lymphedema, significant fibrosis often diminishes the effectiveness of compression pressure before it can adequately reach the edematous tissues, rendering high-pressure compression therapy uncomfortable and frequently necessary (55). However, due to the extensive disruption of tissue involved, complications such as poor wound healing, infections, hypertrophic scarring, and aesthetic changes are common, making the Charles procedure a treatment of last resort for patients with end-stage lymphedema who present with severe skin and subcutaneous fibrosis (56).

In 2007, Salgado et al. (57) presented Radical Reduction with Preservation of Perforators (RRPP), which ensures the conservation of significant vascular perforators and an area of skin flap tissue, while only excising the affected tissue that lies beyond these vital zones. By optimizing the blood supply to the skin flap, RRPP preserves perfusion, eliminates the need for skin grafts, and facilitates thorough resection of compromised tissue, thereby reducing complications. However, RRPP is associated with increased intraoperative bleeding, prolonged operative duration, and necessitates a profound understanding of anatomy and surgical proficiency. Nevertheless, with the advent of RRPP, which minimizes skin trauma and decreases complication rates, RRPP may become the preferred method for severe cases. Recent research suggests that integrating RPPP with physiological techniques could enhance physiological drainage and yield improved long-term outcomes, presenting a promising strategy for the ongoing management of advanced lymphedema.

## Liposuction in lymphedema treatment

Since the introduction of liposuction(LS) for the treatment of upper extremity lymphedema by O’Brien et al. in 1989 (58), this technique has emerged as a preferred minimally invasive option compared to the Charles procedure, which typically requires a total excision of the affected skin. This method minimizes tissue damage and reduces the likelihood of complications commonly associated with more invasive excisional procedures. Unlike physiological surgeries that aim to restore lymphatic drainage, LS is specifically designed to remove excessive fibrotic adipose tissue in cases of chronic, non-pitting lymphedema characterized by fat hypertrophy (59). The prevailing perspective is that LS does not directly facilitate the restoration of lymphatic flow. However, a 2017 study noted instances of lower extremity lymphedema, in which lymphoscintigraphy revealed the regeneration of linear lymphatic vessels located in the medial thigh area following LS treatment (60). Despite the potential of LS to promote lymphatic regeneration, outcomes can vary significantly among patients (61). Consequently, it is essential to continue post-operative compression therapy to maintain results and reduce the risk of recurrence (62).

While LS offers several benefits, it also presents certain risks. Inadequate retraction of postoperative skin redundancy may result in dead spaces, which increase the likelihood of hematoma, skin ischemia, and necrosis. To mitigate these issues, immediate excision of excess skin is recommended. Furthermore, there are persistent concerns regarding LS’s potential to damage subcutaneous lymphatic vessels, which could disrupt lymphatic circulation. The presence of dense fibrotic tissue in hypertrophic adipose tissue complicates fat extraction, often leading to significant intraoperative bleeding (63). To address these challenges, it is advisable to employ specialized LS cannulas and equipment, as well as techniques such as wet LS, longitudinal suction along the limb’s axis, to reduce the risk of iatrogenic lymphatic injury and bleeding (64). In conclusion, LS is an effective and minimally invasive debulking method that can significantly enhance limb function and QOL for patients with advanced lymphedema (65). However, since LS does not resolve the underlying issue of lymphatic obstruction, achieving sustainable therapeutic success depends on ongoing compression therapy or the combination of LS with physiological methods.

## Component-based integrated surgical management of lymphedema

Several internationally recognized centers specializing in the management of lymphedema have proposed diverse treatment strategies based on their clinical expertise (66, 67). Although no universal consensus has yet been established, these strategies generally converge on three key determinants: the severity of lymphedema, the dominant pathological tissue composition, and the functionality of lymphatic drainage (68–70). Multiple classification systems have been developed to evaluate disease severity, among which the International Society of Lymphology (ISL) staging is the most widely applied in clinical practice. The ISL system categorizes lymphedema into four stages: Stage 0 (subclinical, with no visible edema), Stage I (spontaneously reversible edema that subsides with limb elevation), Stage II (spontaneously irreversible edema with associated fibrosis), and Stage III (lymphostatic elephantiasis with marked fibrotic and dermal changes) (71–73). Additionally, ISL guidelines recommend quantifying the volume discrepancy between affected and contralateral limbs to assess functional impairment, classifying severity as mild (<20%), moderate (20–40%), or severe (>40%), which can inform the selection of debulking or physiologic surgical interventions. Importantly, surgical planning should be tailored to the predominant pathological tissue—categorized broadly as fluid-rich (due to lymph stasis and inflammation) or solid-rich (associated with adipose hypertrophy and fibrosis). Accurate differentiation between these components is critical for optimal surgical decision-making. Currently, initial evaluation primarily relies on clinical history and physical examination. For instance, diurnal variation of swelling (worse in the evening) suggests a fluid-dominant phenotype, whereas persistent non-pitting edema (indentation <6–7 mm), papillomatosis, verrucous hyperplasia, and hyperkeratosis are indicative of fibrofatty deposition (74, 75). Imaging modalities can further aid in this distinction; computed tomography (CT) offers reliable differentiation between fluid and fat components (76), while magnetic resonance imaging (MRI) provides superior soft tissue contrast without radiation exposure, albeit with higher costs and longer examination times (77, 78).

In patients with lymphedema primarily composed of fluid-rich components, physiologic surgical interventions have demonstrated substantial clinical value, particularly when CDT is poorly tolerated or insufficiently effective. By actively facilitating lymphatic drainage, these procedures can significantly reduce fluid accumulation and subsequently lessen the patient’s dependence on long-term compression therapy. Among these, LVA is typically considered the first-line option due to its minimally invasive nature and rapid therapeutic response. The effectiveness of LVA can be further enhanced by increasing the number of anastomoses and targeting different lymphatic territories to optimize flow dynamics and bypass obstruction (13–15, 79). Despite these advantages, concerns remain regarding its long-term efficacy, as reduced intralymphatic pressure postoperatively may lead to anastomotic occlusion or venous reflux, ultimately impairing clinical outcomes (23). In cases where LVA yields favorable results, patients frequently exhibit marked reductions in limb volume and symptomatic relief, and some may benefit from staged repeat LVA procedures to consolidate outcomes. Conversely, suboptimal response to initial LVA may reflect poor functional lymphatic reserve, necessitating the consideration of VLNT as a secondary approach (80, 81). In selected cases—particularly those with recurrent cellulitis or lymphangitis—VLNT may serve as a primary treatment modality due to its immunomodulatory properties and capacity to reconstruct lymphatic pathways (39, 82–84). A combined surgical approach, integrating both LVA and VLNT, has shown promise in achieving both immediate and sustained therapeutic benefits (85). VLNT may serve as a foundation for improving regional lymphangiogenesis, which can then be supplemented by LVA to enhance drainage efficiency and reduce limb volume further (7). However, failure to achieve improvement after initial VLNT may suggest poor lymphangiogenic capacity or inadequate recipient bed preparation, which can limit the efficacy of subsequent LVA procedures (86, 87). The distinct mechanisms and therapeutic timelines of these interventions warrant careful consideration. LVA establishes direct lymph-to-vein shunting and offers immediate symptomatic relief, whereas VLNT relies on gradual processes such as lymphangiogenesis and nodal pumping, typically requiring up to six months to achieve optimal results. Notably, while LVA demonstrates superior short-term benefits, VLNT may provide more durable long-term outcomes (6). Therefore, for patients with fluid-predominant lymphedema, physiologic surgery represents a preferred therapeutic paradigm. However, individualized treatment planning—potentially involving the strategic combination of LVA and VLNT—is essential to maximize clinical efficacy and minimize the need for repeat surgical interventions.

**Figure 2:**
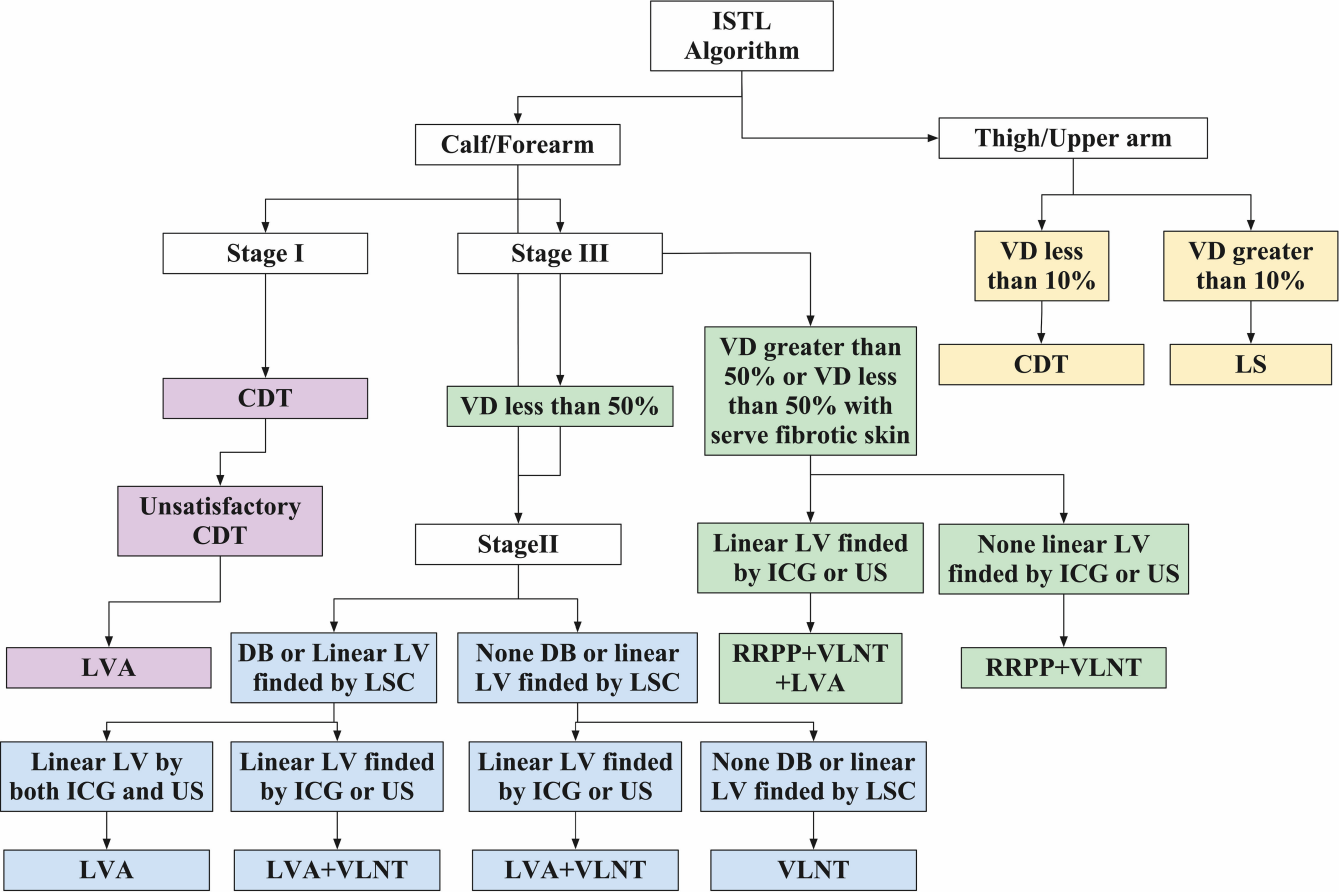
ISTL algorithm for SEL. CDT, comprehensive decongestive therapy. LVA, lymphaticovenous anastomosis; LV, lymphatic vessels; ICG, indocyanine green; US, ultrasound; VD, volume difference; LSC, lymphoscintigraphy; VLNT, vascularized lymph node transfer; RRPP, radical reduction with preservation of perforators; LS, liposuction.

**Figure 3:**
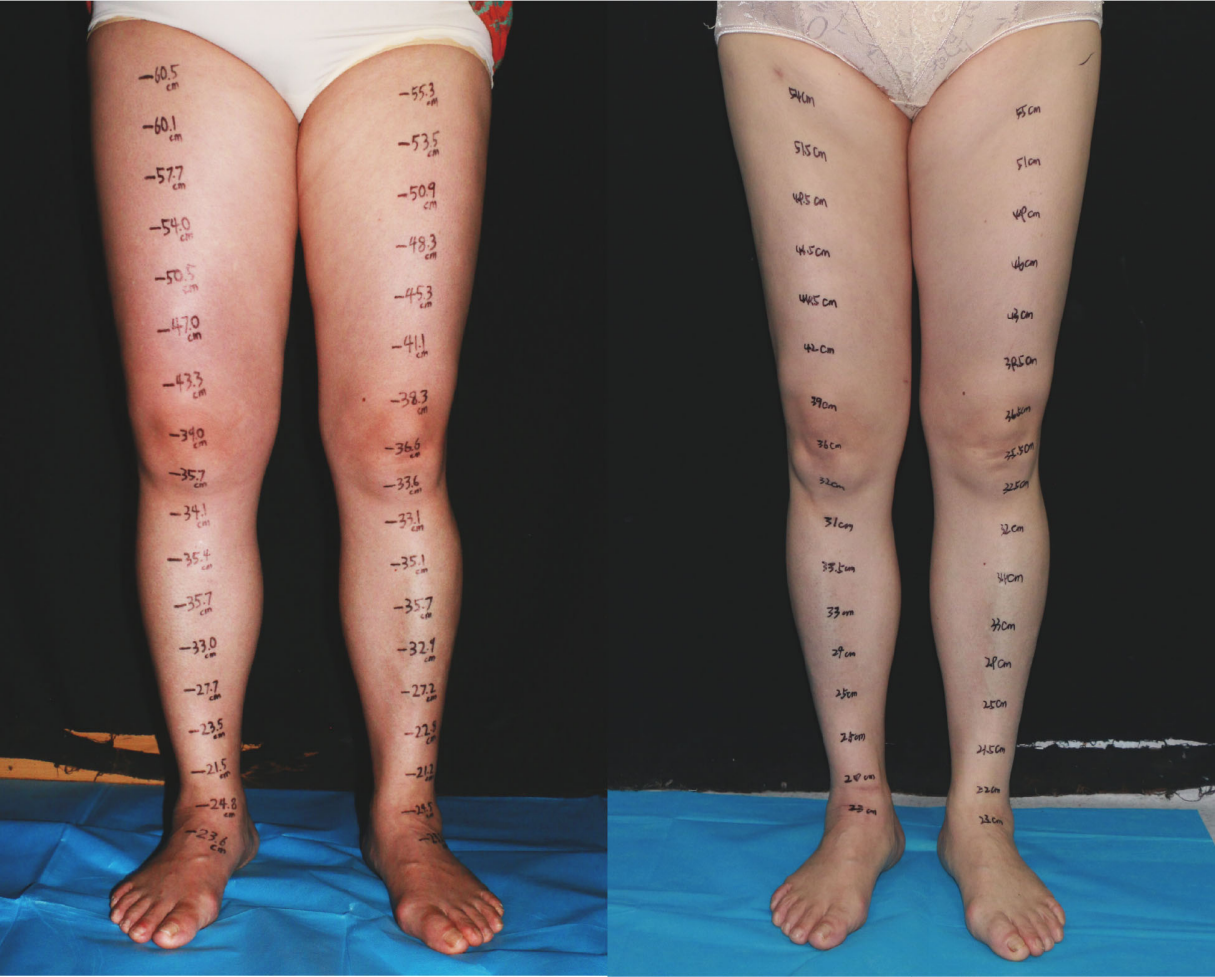
A 57-year-old female patient with ISL stage 2 lymphedema of the right lower limb is shown (left). The edema progressed over a 2-year period. The patient underwent three end-to-end lymphaticovenous anastomoses at ankle level and liposuction in the thigh. The patient at post-surgery 20 months shows complete reduction matching the contralateral normal limb(right).

**Figure 4:**
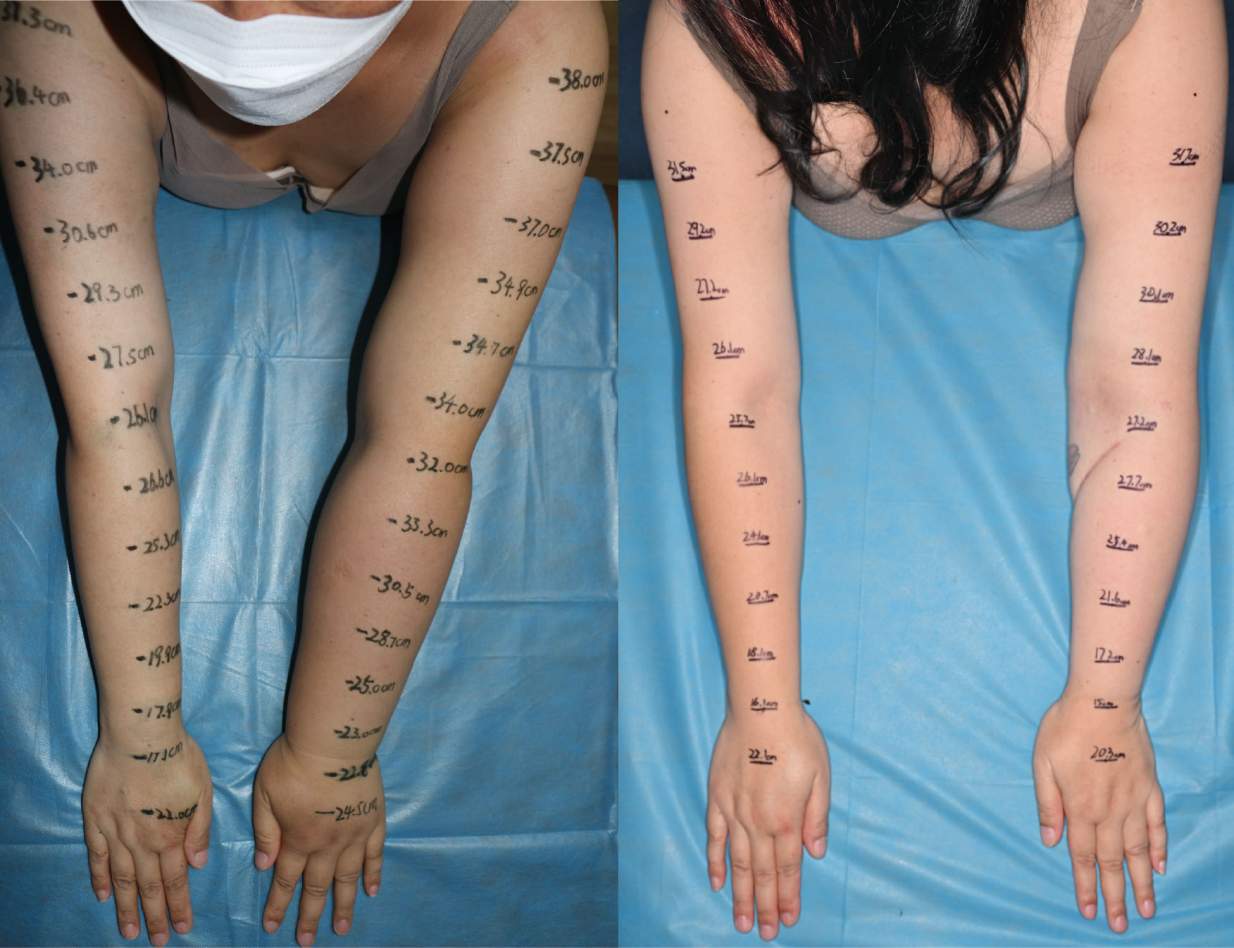
A 47-year-old female patient with ISL stage 2 lymphedema of the left upper limb is shown(left). The edema progressed over a 3-year period. The patient underwent supraclavicular vascularized lymph node transfer at elbow fossa level and liposuction in the upper arm. The patient at post-surgery 30 months shows significant reduction (right).

In patients with lymphedema characterized by predominant solid components—mainly due to extensive adipose hypertrophy and fibrosis—the excessive pathological tissue significantly impairs limb mobility and, in severe cases, may render patients unable to stand or ambulate. At this stage, reductive surgery becomes essential to reduce the mass effect and improve functional capacity and quality of life. However, such procedures do not address the underlying lymphatic drainage dysfunction, and the limb often reaccumulates lymph fluid postoperatively, leading to continued or even increased dependence on compression garments and conservative therapy. Therefore, combining debulking procedures with physiologic surgeries—which aim to restore lymphatic continuity—has been increasingly recommended to sustain volume reduction and reduce the risk of recurrence. The choice of combined strategy should be tailored to the dominant solid component. In patients with predominantly adipose tissue proliferation, LS combined with physiologic procedures such as LVA or VLNT is preferred (**Figures 2**, **3**). For those with advanced elephantiasis, characterized by severe fibrotic and dermal thickening, more aggressive excisional approaches such as the Charles procedure or RRPP, combined with physiologic reconstruction, may be required. Although LS alone can significantly reduce limb volume, and some reports suggest spontaneous restoration of linear lymphatic flow following LS, current consensus emphasizes that LS should be complemented by physiologic surgery to minimize the risk of relapse and reduce reliance on CDT (60, 61). Due to the potential for LS to disrupt remaining functional lymphatics—and the difficulty of identifying them intraoperatively—selective LS techniques have been proposed. These involve preserving areas with preoperatively mapped lymphatic vessels, allowing physiologic procedures to be performed either during the same session (single-stage) or at a later time (staged) (88, 89). Some studies further suggest that total resection of lymphatic structures above the deep fascia followed by subfascial LVA can still yield satisfactory outcomes (90). In advanced-stage disease where functional lymphatic vessels are sparse or unidentifiable, LS combined with VLNT has emerged as a superior approach (91–96). Moreover, 3L procedures, integrating LS, LVA, and VLNT (**Figure 4**), may provide enhanced benefits compared to traditional 2L strategies(LS+LVA and LS+VLNT). Evidence suggests that 3L surgery results in greater limb volume reduction, decreased skin tension, and a lower incidence of infections such as cellulitis (97). Nonetheless, clinical data on 3L surgery remain limited, and prospective randomized controlled trials are needed to validate its superiority and clarify its indications. For patients undergoing RRPP due to extensive fibrosis, preoperative identification of functional lymphatics may allow for the concurrent performance of LVA to restore drainage pathways (98). However, in many cases of late-stage lymphedema, chronic inflammation and recurrent infections—such as cellulitis and lymphangitis—compromise the efficacy of LVA. In such settings, RRPP or Charles surgery combined with VLNT may offer superior outcomes by simultaneously reducing the fibrotic mass and restoring immune and lymphatic function in the affected limb (99, 100).

Although the combination of reductive and physiologic surgical approaches has demonstrated encouraging outcomes in the management of lower extremity lymphedema, several challenges remain. These include prolonged operative time, technical complexity, and increased surgical trauma compared to single-modality procedures. Furthermore, several controversies persist—particularly regarding the optimal timing of combined interventions. The choice between single-stage versus staged surgical strategies has emerged as a topic of active investigation. Staged approaches aim to address the predominant pathological component first to achieve symptom relief. For patients with solid-dominant disease, debulking procedures are typically prioritized, whereas physiologic reconstruction is favored in fluid-predominant cases (101). Staged surgery also offers potential advantages, such as the possibility of avoiding a second operation if the initial procedure achieves sufficient clinical improvement, thereby minimizing overall surgical trauma. In contrast, for patients with mixed pathology—i.e., substantial fibroadipose tissue and fluid retention—a single-stage combined procedure is often employed to address both components simultaneously. One-stage surgery also has the benefit of reducing the need for rehospitalization, alleviating both economic burden and time costs for the patient. However, technical considerations must be carefully addressed when planning one-stage procedures. LVA requires preservation of functional lymphatic vessels, and VLNT demands adequate vascular supply and minimal early postoperative compression to ensure graft viability. Performing volume-reductive procedures in the same anatomical field may compromise these physiological requirements and potentially blunt the therapeutic effect (102). To mitigate these risks, the implementation of selective debulking techniques and anatomical compartmentalization of surgical fields has been proposed. This strategy involves performing debulking and physiologic surgeries in separate anatomical zones, preserving critical lymphatic structures and vascular networks. Moving forward, the development of standardized surgical protocols, including decision-making algorithms for combined staging, tissue-specific treatment pathways, and perioperative management guidelines, will be essential to optimize clinical outcomes and reduce variability across institutions.

## Our algorithm

**Figure 5:**
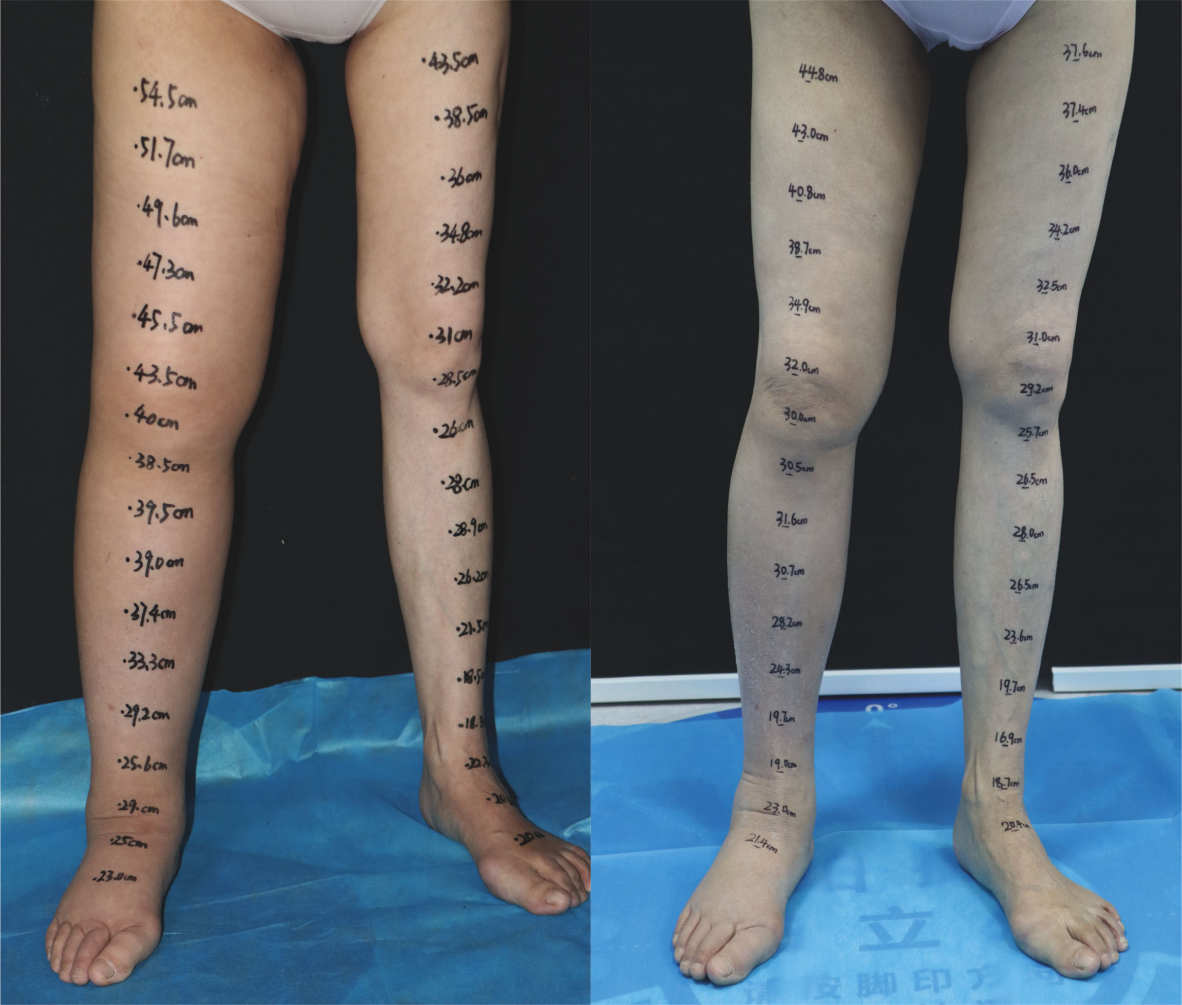
A 54-year-old female patient with stage 3 lymphedema of the left lower limb is shown (left). The edema progressed over a 5-year period. The patient underwent three end-to-end lymphaticovenous anastomoses at ankle level, supraclavicular vascularized lymph node transfer at popliteal fossa and liposuction in the thigh. The patient at post-surgery 32 months shows complete reduction matching the contralateral normal limb (right).

**Figure 6:**
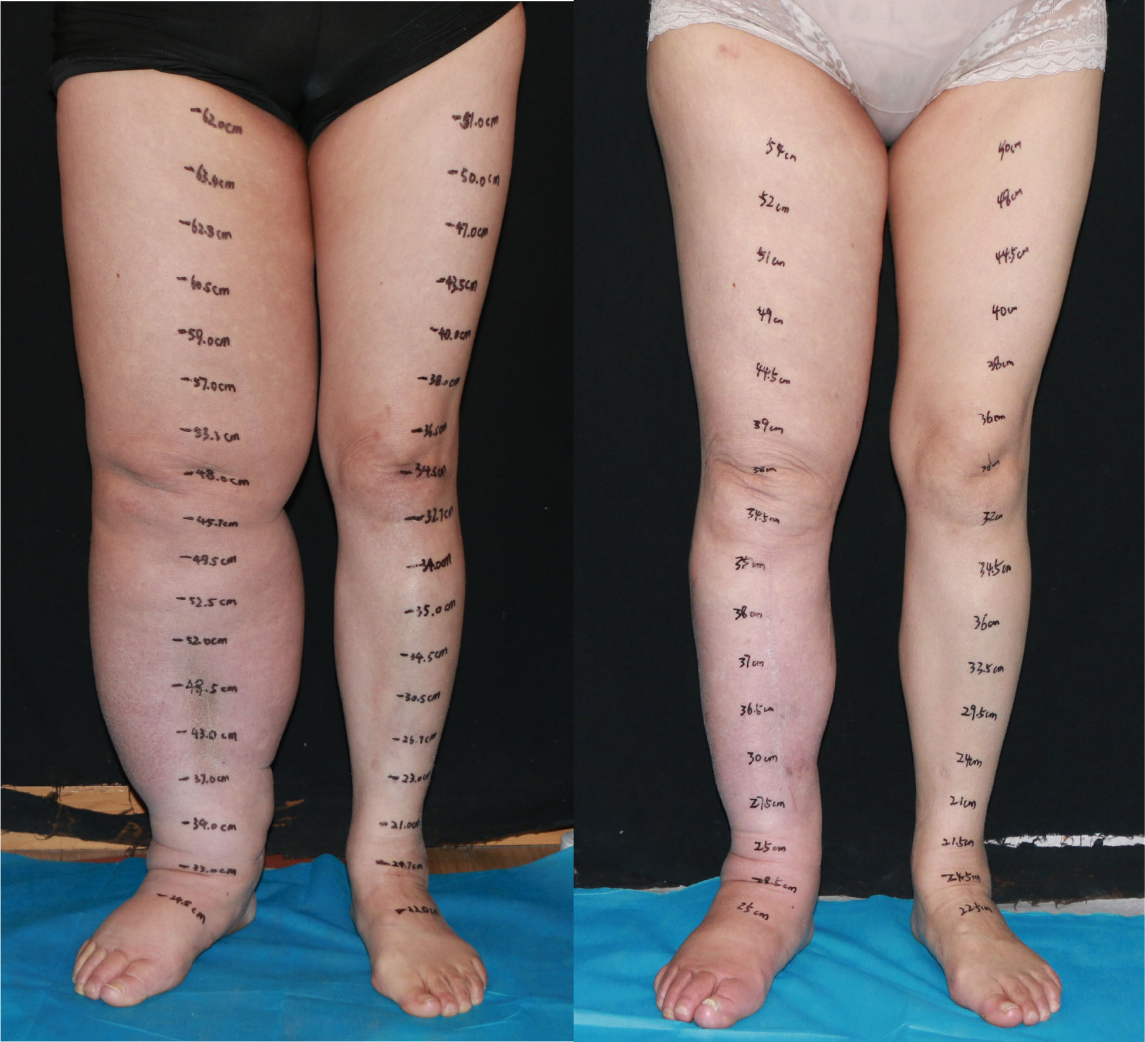
A 56-year-old female patient with skin change, minimal pitting, and advanced fibrosis is shown with stage 3 lymphedema of the left lower limb is shown (left). The edema progressed over an 8-year period. The patient underwent radical reduction with preservation of perforators in the calf, supraclavicular vascularized lymph node transfer at popliteal fossa and liposuction in the thigh. The patient at post-surgery 34 months shows significant reduction (right).

Significant advancements have been made in lymphedema surgery; however, selecting appropriate procedures remains critical to achieving optimal outcomes, particularly in the absence of standardized treatment guidelines (68, 102, 103). To address this, our center developed the ISTL(Integrated Surgical Treatment of Lymphedema) algorithm (**Figure 2**), which tailors surgical strategies based on volume difference (VD), disease stage, anatomical location, lymphatic vessel function, and clinical presentation. Patients are first stratified by the affected region—thigh/upper arm or leg/forearm—with region- and stage-specific protocols. In proximal limb involvement (thigh/upper arm), thick fibrotic adipose tissue often obscures functional lymphatic vessels (FLV), limiting the efficacy of LVA; thus, LS is preferred. Patients with VD <10% are managed with CDT, while those with VD ≥10% are directed to LS (75, 104–106). For distal limb lymphedema (leg/forearm), treatment aligns with International Society of Lymphology (ISL) staging. In Stage I, LVA is indicated if CDT is insufficient. In Stage II, treatment depends on lymphatic imaging: if lymphoscintigraphy(LSG) reveals discrete or linear lymphatics and these are confirmed by ICG or ultrasonography (US), standalone LVA is preferred (**Figure 3**) (107, 108); if linear lymphatics are not identified on ICG or US, combined LVA and VLNT is recommended; if LSG, ICG, and US all show no linear vessels, VLNT alone is indicated (**Figure 4**); however, if ICG or US detects linear vessels despite negative LSG, combined LVA and VLNT is preferred (**Figure 5**). In Stage III, treatment is dictated by VD and skin condition: patients with VD <50% follow Stage II protocols, while those with VD ≥50% or with severe skin fibrosis require aggressive intervention, such as RRPP with VLNT (**Figure 6**) (109). Notably, if any functional LV is present, a comprehensive approach combining RRPP, VLNT, and LVA is favored to optimize long-term outcomes. This comprehensive algorithm integrates clinical evaluations, imaging diagnostics, and surgical interventions—including LVA, LS, VLNT, and RRPP—to deliver personalized, stage-specific therapies for patients affected by lymphedema.

Although this study presents a systematic and reproducible treatment algorithm for lymphedema, several limitations merit further discussion. First, while the imaging and treatment thresholds were established based on high-quality clinical studies, their applicability may vary across clinical settings due to differences in available resources, equipment, and microsurgical expertise. Thus, despite its broad potential applicability, individualized treatment planning remains essential. Although imaging modalities such as CEUS, ICG lymphography, and lymphoscintigraphy are widely available in many tertiary centers, their use may be constrained in some institutions by technical limitations, operator experience, and imaging infrastructure, thereby affecting widespread implementation. Additionally, while the algorithm is designed to address moderate and severe lymphedema, it does not fully incorporate individual patient-level factors—such as age, sex, BMI, or comorbidities—that may significantly impact treatment selection and outcomes. Future studies should explore these variables in greater depth to further personalize and refine the algorithm. Moreover, this study is based primarily on retrospective data and previously published literature. Although rigorous data screening and quality control were applied, the potential for selection bias cannot be excluded, and the external validity of our findings requires confirmation through prospective, randomized controlled trials. In light of these considerations, we emphasize that the algorithm is intended as a practical, feasibility- and reproducibility-oriented guide, not a rigid or exclusionary framework. For example, proximal LVA is not excluded within this system. When preoperative imaging identifies accessible deep collecting lymphatics and appropriate microsurgical expertise is available, proximal LVA remains a feasible option. In practice, two proximal pathways are recognized: (1) superficial proximal LVA, which may be limited by fibroadipose overgrowth, sparse venules, and poor ICG visualization, making intraoperative identification more difficult (110); and (2) deep proximal LVA, which becomes feasible when deep anatomy is favorable. However, this technique presents additional technical challenges—such as greater operative depth, loss of ICG linearity, reduced effectiveness of UHFUS, and the frequent need for antegrade end-to-side anastomosis (LVESA) using a back-wall-first technique due to LV–vein caliber mismatch. In cases of limited recipient veins, multiple LVESAs on a single vein within a confined space may be necessary, and higher BMI may further complicate exposure (111). Given these complexities, in patients with proximal disease, we often prioritize liposuction for initial volume reduction, followed by reassessment for physiologic reconstruction. Notably, liposuction has been shown to improve lymphatic function and promote lymphatic remodeling, as reported in the New England Journal of Medicine (60). In contrast, distal segments of the limb more consistently demonstrate functional lymphatics on ICG and ultrasound, and offer more recipient venules, making distal LVA a more reproducible and preferred strategy in our algorithm. Regarding VLNT, proximal inset is not excluded either. The optimal inset site remains a topic of ongoing research, with successful outcomes reported for proximal, mid-limb, and distal placements. In our practice, calf or peri-knee insets are often favored for lower-limb VLNT, leveraging the joint’s mechanical “pump” function—conceptually similar to the “Seki point”—which may facilitate lymphatic return (112–114). In these positions, VLNT may activate both the “pump” and “bridge” mechanisms of lymphatic drainage.

The approach to managing surgical interventions for SEL has undergone significant advancements, evolving from standalone surgical methods to personalized strategies that integrate physiological techniques with excisional surgery. However, several pressing challenges remain. Ongoing research and standard algorithm are essential for enhancing surgical decision-making and achieving optimal, durable outcomes for individuals with SEL.
